# Study on the Influence Mechanism of Virtual Simulation Game Learning Experience on Student Engagement and Entrepreneurial Skill Development

**DOI:** 10.3389/fpsyg.2021.772157

**Published:** 2022-01-27

**Authors:** Qixing Yang, Yue Zhang, Yawen Lin

**Affiliations:** Zhongshan Institute, University of Electronic Science and Technology of China, Zhongshan, China

**Keywords:** virtual simulation game, learning experience, student engagement, entrepreneurial skills development, 3P model

## Abstract

With the emergence of the COVID-19 pandemic, virtual simulation games have provided an effective teaching method for online entrepreneurship education. By exploring the mechanisms that influence student engagement and learning outcomes from different perspectives, such as game design, team and individual perspectives, numerous scholars have demonstrated that such a teaching method can effectively improve students’ engagement and learning performance. However, the existing studies are relatively scattered, and there is a scarcity of studies in which the effects of said factors are considered. Based on the learning process 3P model (presage-process-product) proposed by Biggs (1993), students’ perceived experience of game design, teamwork and self-efficacy were taken as variables in the early learning stage in the present study, and the influence mechanism of virtual simulation game learning experience on students’ engagement and entrepreneurial skill development was explored, so as to close the gap in existing research. In the present study, 177 college students from Chinese universities were surveyed and the data were surveyed using AMOS 23.0 software. Although the empirical results show that students’ “goal and feedback” and “alternative” experience of game design did not have a significant positive impact on students’ engagement, there was a direct and significant effect the development of entrepreneurial skills. Students’ experience of teamwork and general self-efficacy could not only directly and significantly affect the development of entrepreneurial skills, but also indirectly affect the development of entrepreneurial skills through learning engagement. The research results are practically significant for teachers in the selection and development of virtual simulation games, can be effectively applied in teaching process management, and can improve students’ engagement and learning performance.

## Introduction

In recent years, determining how to improve college students’ learning experience of entrepreneurship education and achieve better results of entrepreneurship learning has become a trending topic in the field of entrepreneurship education (Fayolle, 2013; Yang et al., 2021). Academics have introduced a variety of notable teaching methods and tools into the curriculum, with simulation games being one of the most common. Through a 10-year follow-up survey of German students who participated in a start-up simulation competition, Kriz and Auchter (2016) confirmed that start-up simulation games can significantly improve students’ business management knowledge and business plan preparation skills. Charrouf and Janan (2019) adopted a game to simulate real business operation in entrepreneurship courses, which improved students’ engagement and achieved good teaching effects. Results of a survey of 180 teachers in Europe showed that simulation games can improve students’ participation in the classroom, enhance students’ communication and cooperation in the classroom, and improve their knowledge and abilities (Mirjana et al., 2020). Isabelle (2020) also adopted gamified teaching methods for 269 college students in the course of entrepreneurship, which improved students’ experience level, engagement and entrepreneurial efficacy. As confirmed by the latest study by Zulfiqar et al. (2021), business simulation games can significantly increase students’ investment and acceptance of entrepreneurship courses, thereby significantly improving entrepreneurial intention and learning performance. An observation can be made that the effectiveness of the teaching method of simulation games for students’ entrepreneurial learning has been confirmed by the majority of scholars. To determine the process of implementing such a teaching method, the that factors will affect students’ participation and learning effect, and the mechanisms of influence, scholars mainly conducted research from the following three directions:

The focus of the first direction has been on simulation games themselves. Almeida and Buzády (2019) adopted serious games in an entrepreneurship course and found, through focus group interviews, that students were particularly concerned about the authenticity, interactivity and feedback of the system. Based on Flow theory, through empirical research, Yen and Lin (2020) found that challenge-skill balance and playability of simulation games significantly positively affected players’ Flow experience. However, goals, feedback and control had no significant positive impact on flow experience, and flow experience had a significant positive impact on perceived learning performance of players, which in turn affected entrepreneurial self-efficacy. Predicated on the technology acceptance model (TAM), through empirical research, Zulfiqar et al. (2021) found that perceived usefulness and perceived ease of use affected students’ adoption of the simulation game system, and then significantly positively affected entrepreneurial intention and learning performance. Capelo et al. (2021) believed that exposing the model in the “black box” of business simulation game to students would help students to understand the dynamic relationship between variables and improve the performance level of students’ simulation.

The focus of the second direction has been on teamwork between students participating in the game. In simulation game courses, team members need to trust each other and communicate openly, which can expand their knowledge, and improve decision-making accuracy (Thanasi-Boçe, 2020). As confirmed by existing studies, students’ satisfaction with team member relationships will affect their learning engagement, and then affect perceived learning gain and skill development (Buil et al., 2020). Team cooperation will also directly and significantly positively affect the results and satisfaction of business simulation learning (Lohmann et al., 2018). In the simulated decision-making process, although opinions among team members may not always be consistent, even team conflict has been demonstrated to significantly improve students’ entrepreneurial attitude, and students’ ability has also been trained in the process of managing team conflict (Arias-Aranda and Bustinza-Sánchez, 2009).

The focus of the third direction has been on the idiosyncratic aspects of playing games. The empirical research results of Buil et al. (2020) revealed that students’ satisfaction with competence, independent choice, membership and self-efficacy will affect their engagement in business simulation games, and then affect students’ skill development and knowledge acquisition. Hernández-Lara et al. (2019) confirmed that generic skills such as teamwork, decision-making ability and information processing ability of students participating in simulation games had a significant positive impact on learning outcomes. However, special management skills (such as strategic management ability, financial data processing and analysis ability, risk management ability, project management ability, and others.) had no significant impact on learning results, in addition to students’ previous course scores (Alstete and Beutell, 2019), whether they usually play video games (Charrouf and Janan, 2019), and critical thinking (Eggers et al., 2017).

Reviewing the existing literature, an observation can be made that the effectiveness of business simulation games in entrepreneurship education has been confirmed by a large number of studies (Kriz and Auchter, 2016; Charrouf and Janan, 2019; Isabelle, 2020; Mirjana et al., 2020; Zulfiqar et al., 2021); however, there are still a number of significant deficiencies in existing research on how to improve students’ participation and learning effect in the process of implementing such teaching method.

First, studies on antecedents affecting students’ learning engagement are scattered. Some studies have focused on the impact of games themselves on learning outcomes (Almeida and Buzády, 2019; Yen and Lin, 2020; Capelo et al., 2021; Zulfiqar et al., 2021), some have focused on teamwork (Arias-Aranda and Bustinza-Sánchez, 2009; Lohmann et al., 2018; Buil et al., 2020; Thanasi-Boçe, 2020), and others have focused on students’ personal traits (Eggers et al., 2017; Alstete and Beutell, 2019; Charrouf and Janan, 2019; Hernández-Lara et al., 2019; Buil et al., 2020). No study has taken into account the characteristics of games, teams and individuals.

Second, there are limitations in the selection of theoretical framework. Most existing studies have been based on the planned behavior theory (Newbery et al., 2016; Zulfiqar et al., 2018, 2021), the technology acceptance model (Zulfiqar et al., 2018, 2021), and Flow theory (Choi and Kim, 2004; Yen and Lin, 2020). Through such frameworks, there is a tendency to limit the perspective to the game system itself, and there is a failure to fully present the complete chain of “input-process-result” and its mechanism of entrepreneurial simulation learning.

Third, there is a scarcity of studies on the learning effect of entrepreneurial simulation games from the perspective of student engagement. Despite students being the main body in the process of entrepreneurial learning, most existing studies have confirmed the effectiveness of the simulation game teaching method, and few have considered the factors that will affect students’ engagement from the perspective of students.

In the present study, based on Biggs (1993) ’s presage-process-product (3P) model, games, teams, and individuals were considered as pre-learning variables, student engagement was taken as the learning process variable and entrepreneurial skill development was taken as the learning outcome variable. The formation mechanism of student engagement and learning outcome was explored. In theory, of the present study can close the gap in existing research. The research results can also provide significant inspiration for teachers and simulation game developers who conduct entrepreneurial simulation experiment courses, and have considerable practical significance.

## Theoretical Review and Research Hypothesis

### Virtual Simulation Games

In 1957, Professor Schreiber of University of Washington developed a simulation game named “Top Management Decision Game,” which was introduced into the curriculum (Watson, 1981). Since then, the teaching method has been extensively adopted in business majors, especially after the 1980s. Amongst the background of computer popularization, computer simulation games have been rapidly popularized and applied in management courses, and a large number of scholars have conducted relevant studies. Over 1200 related papers were published between 1960 and 2019 alone (Hallinger and Wang, 2020). Virtual simulation games have been based on the experiential learning theory (Kolb, 1983), combined with the organization theory and the game theory, to design rules and algorithms (Sterman, 1994; Geurts et al., 2007), which is widely used in business education (Keys and Wolfe, 1990; Wolfe, 1993; Anderson and Lawton, 2009; Faria et al., 2009). The teaching method of virtual simulation game has also been recently applied in entrepreneurship education in many colleges and universities, and has been demonstrated to effectively improve the investment and learning performance of college students in entrepreneurship learning (Kriz and Auchter, 2016; Charrouf and Janan, 2019; Isabelle, 2020; Zulfiqar et al., 2021).

In the present study, a virtual simulation game was used in the course of entrepreneurship education. Students participated in the course in teams of 3–5 people. Each team needed to register and set up a company, study the market demand, clarify the priority market segments for the team to enter, position the style and characteristics of the product, and decide the research and development costs. Each virtual company needed to decide whether to build its own factory and the size of the factory, as well as the financing method. The required funds could be obtained through bank loans, issuing bonds and selling shares. On a quarterly basis, each company needed to make financial budgets and decisions on 62 projects, covering research and development, marketing, production, logistics, human resources and other aspects of business operations. Sixteen companies were allowed to compete in each industry, and at the end of each quarter, the system’s mainframe ran calculations based on the decisions submitted by each company and reported the results of the game back to each company. At least eight quarters of simulation were conducted in each round.

### Learning Process 3P Model

Biggs (1993) proposed the presage-process-product (3P) model of college learning. In said model, the early variables include students’ individual characteristics and learning experience, the process variables mainly refer to students’ learning methods, and the outcome variables refer to students’ performance and gains. Prophase variables determine how students deal with a certain task, which further affects their learning results. That is, learning style is a mediating factor between learning experiences and learning results. Simultaneously, learning experiences directly predict learning outcomes (Trigwell et al., 2013). The interaction between prophase variables, process variables and outcome variables forms a dynamic system (Biggs et al., 2001).

The learning result of college students is a key indicator in evaluating the quality of university education (Douglass et al., 2012). According to existing studies, factors affecting the learning result can be essentially classified into two categories: student engagement (Fredricks et al., 2004) and learning experience (Richardson, 2005). Numerous studies have confirmed that student engagement of college students is a significant predictor of learning outcomes. Academic achievement and satisfaction will be higher with the investment of more time, energy and emotion from students (Wang and Eccles, 2013; Zepke, 2014; Zusho, 2017). College students’ learning experience refers to their perception, opinion and understanding of the learning environment (Entwistle, 1991). A large number of studies have shown that college students’ perception of the learning environment can affect their learning behavior and learning results (Diseth, 2007; Trigwell et al., 2013; Guo et al., 2017). However, there is a scarcity of existing studies in which the relationship between learning experience, learning engagement and learning outcomes is explored (Yin and Ke, 2017), especially in the field of entrepreneurship education. As such, in the present study, the 3P model proposed by Biggs (1993) was taken as the research framework, students’ learning experience in virtual simulation game courses was taken as the independent variable, students’ engagement was taken as the intermediary variable, and entrepreneurial intention was taken as the dependent variable to construct the research model.

### Virtual Simulation Game Course Learning Experience

Learning experience is the main factor affecting student engagement (Astin, 1984; Biggs, 1993; Coates and McCormick, 2014), in order to improve the level of students’ learning experience, the teaching content needs to be designed and the teaching process needs to be managed with students as the center of the concept (Gibb, 2002; Murah and Abdullah, 2012; Robinson et al., 2016).

Virtual simulation games are first and foremost games, so the experience of the game itself is a significant factor that constitutes students’ course experience. Choi and Kim (2004) divided the influencing factors of the online game player experience into human-computer interaction factors and social interaction factors. Human-computer interaction includes goals, feedback and operability, while social interaction includes interaction place and interaction way. Such factors have been confirmed to have a significant positive impact on player experience. Yen and Lin (2020) introduced a retail simulation game into a marketing course to build a research model based on Flow theory. The antecedents of flow experience were divided into challenge-skill balance, playability, goals, feedback and control, and the influence of such variables on flow experience and learning performance was investigated.

In the course of virtual simulation games, students usually participate in a competition in the form of a team. As such, the communication, trust and cooperation between team members will certainly affect students’ investment and learning performance. Thanasi-Boçe (2020) adopted a marketing simulation system in a course. Sixteen students were divided into four teams to conduct a simulated competition. Through qualitative investigation and research, students gave feedback that team cooperation was crucial, and the interaction and even conflict among team members was found to have improved their ability to manage the team. Lohmann et al. (2018) conducted a survey of 365 students in Australia and Hong Kong and confirmed that teamwork can directly and significantly positively affect students’ results and satisfaction with business simulation learning.

Obviously, the personal characteristics of participants in virtual simulation games are also significant factors that can affect learning engagement and performance. Buil et al. (2020) constructed the self-system model of motivational development (Skinner et al., 2008). Based on the survey results of 360 students, the satisfaction degree of competency, independent choice, membership and self-efficacy was found to affect their cognitive, affective and behavioral engagement in business simulation games, and the cognitive and affective engagement would significantly positively affect students’ skill development and knowledge acquisition. Hernández-Lara et al. (2019) surveyed 115 undergraduate and master’s students and found that, in business simulation game courses, students’ generic skills (such as teamwork, decision-making ability, information processing ability, entrepreneurial ability, and new technology application ability) could have a significant positive impact on learning outcomes.

To summarize, the following hypothesis is proposed in the present study:

H1: Learning experience in virtual simulation game courses includes game experience, team experience and self-efficacy.

### Learning Experience and Student Engagement and Entrepreneurial Skills Development in Virtual Simulation Game Courses

Student engagement is a multidimensional concept, including students’ time, energy and investment in cognition, emotion and behavior. Cognitive investment refers to the use of deep learning methods and strategies, with intrinsic learning motivation, emotional investment refers to the interest and satisfaction of learning and the relationship with teachers and peers, and behavioral investment refers to the participation in in-class and out-of-class learning activities related to behavior (Fredricks et al., 2004). In general, learning results include increases in knowledge, improvements in ability and changes in attitude (Anderson and Lawton, 2009). Since the focus of the virtual simulation game adopted in the present study was on the application of knowledge and improvement of analytical and decision-making ability, entrepreneurial skill development was measured as an indicator of learning results.

First, good education game design has clear education objectives and performance evaluation standards, and gives feedback and rewards according to students’ performance in the game. Students will strive to achieve performance objectives or gain an advantageous position in the competition. The sense of achievement of breaking through customs and the sense of honor of winning the competition urge students to be willing to invest a lot of time and energy in independent learning. Students will carefully analyze the competitive situation, evaluate the decisions of competitors, seek differentiated products and marketing innovation, reduce operating costs, and ensure sound financial operation. Second, all decisions are made by the student team. Therefore, students’ ability to innovate, analyze and solve problems, and make decisions can be trained and improved. In the course of virtual simulation games, team members trust each other and cooperate closely, which can make participants feel comfortable. Team members enhance each other’s knowledge in interaction. Division of labor and cooperation can improve the efficiency and accuracy of decision-making. Therefore, a good team atmosphere can make students more willing to participate in simulation games, and their ability can be better developed. Third, students’ sense of self-efficacy is also considerably important. Students who are more confident in virtual simulation games are more willing to invest time, energy and emotion to study the rules of the game and competitors, and can gain more. A study by Charrouf and Janan (2019) confirmed that, compared with students who have no experience playing video games, students who like to play video games at ordinary times would find start-up simulation games easier to operate, and would be more willing to devote themselves to learning, with stronger entrepreneurial intention.

As confirmed by a large number of studies, simulated game experience can effectively improve students’ participation and learning outcomes (Anderson and Lawton, 2009; Beltrão and Barçante, 2015; Kriz and Auchter, 2016; Liberona and Rojas, 2017; Charrouf and Janan, 2019; Isabelle, 2020; Mirjana et al., 2020; Kauppinen and Choudhary, 2021; Zulfiqar et al., 2021). Thus, the following hypotheses are proposed in the present study:

H2a: In the virtual simulation game course, game experience has a significant positive impact on student engagement;
H2b: In the virtual simulation game course, team experience has a significant positive impact on student engagement;
H2c: In the virtual simulation game course, self-efficacy significantly positively affects students’ engagement;

H3a: In the virtual simulation game course, game experience has a significant positive impact on the development of students’ entrepreneurial skills;
H3b: In the virtual simulation game course, team experience has a significant positive impact on the development of students’ entrepreneurial skills; and
H3c: In the virtual simulation game course, self-efficacy significantly positively affects the development of students’ entrepreneurial skills.

### Engagement and Entrepreneurial Skills Development of Students in Virtual Simulation Game Courses

In the field of university learning, Astin (1984) paid early attention to the relationship between student engagement and learning results, proposing that students’ learning gains and development are directly related to the quantity and quality of students’ learning input. Numerous subsequent studies have confirmed that student engagement is a significant predictor of learning outcomes, and the more students engage in learning, the better their academic performance will be (Wang and Eccles, 2013; Zepke, 2014; Zusho, 2017). In the field of entrepreneurship education, there is a scarcity of research on the relationship between entrepreneurial learning input and learning outcomes. Several scholars have confirmed that entrepreneurial learning input can improve entrepreneurial self-efficacy (Minniti and Bygrave, 2001; Isabelle, 2020) and entrepreneurial confidence (Rae and Carswell, 2001), thereby influencing entrepreneurial intention. In a recent study, Zulfiqar et al. (2021) introduced business simulation into entrepreneurship courses and confirmed that such teaching method significantly increased student engagement, which in turn significantly improved entrepreneurial intentions and learning performance. As such, the following hypotheses are proposed in the present study:

H4: In the virtual simulation game course, student engagement has a significant positive impact on the development of entrepreneurial skills.

## Research Design

### Data Sources

In the present study, students from the University of Electronic Science and Technology of China, Zhongshan Institute, were taken as the survey object. Students used virtual simulation games in the course, and students formed teams of 3–5 people. The startup teams had to create a new company within the system, develop new products to market, and compete with other teams. After the entrepreneurship course, the research team conducted an electronic questionnaire survey among 192 students who participated in the course. From business major students, 177 valid samples were obtained, including 44% male students and 56% female students.

### Measuring Tools

For the course learning experience, system design, team experience and self-efficacy were comprehensively considered, and the research results of Choi and Kim (2004), Lohmann et al. (2018), and Buil et al. (2020) were comprehensively drawn on to design 13 questions. See Supplementary Appendix QUESTIONNAIRE Q1–Q13 for details. Four questions were designed for student engagement based on the measurement scale of Fredricks et al. (2004) and Buil et al. (2020). See questionnaire Q14–Q17 in the Supplementary Appendix. The research results of Isabelle (2020) was used to form an entrepreneurial skills development scale and four questions were designed. See Supplementary Appendix Q18–Q21 for details. All question types were measured by the 7-level Likert scale, with 1 meaning strongly disagree and 7 meaning strongly agree.

## Results

### Exploratory Factor Analysis of Virtual Simulation Game Course Learning Experience Scale

Exploratory factor analysis was conducted on the learning experience scale using SPSS 23.0 software. The KMO value was 0.861, and the significance level of Bartlett’s sphericity test was lower than 0.001. The number of factors was not limited, and the component matrix after rotation is shown in Table 1. Factor 1 was “goal and feedback” (GF), Factor 4 was “selectivity” (SL), Factor 2 was “teamwork” (TW), and Factor 3 was “general self-efficacy” (GSE). The loads of all items were between 0.628 and 0.904, and the cumulative variance interpretation of all factors reached 79.725%. The overall variance interpretation rate of the first factor was 48.244%, less than 50%. Therefore, the present study was seemingly not affected by the common method deviation. GF and SL were game experiences, TW was team experience and GSE was personal efficacy, and thus, H1 was verified.

**TABLE 1:** Component matrix of learning experience scale after rotation.

Item	Composition			
	1	2	3	4
Q2	0.814			
Q1	0.812			
Q3	0.732			
Q4	0.628			
Q9		0.904		
Q10		0.868		
Q8		0.838		
Q12			0.878	
Q13			0.861	
Q11			0.839	
Q5				0.884
Q6				0.825
Q7				0.677

### Scale Reliability and Validity

On the basis of 177 pieces of valid sample data, the reliability of the scale was tested by SPSS 23.0 software. The Cronbach’s α coefficient of the overall scale was 0.95, and the Cronbach’s α coefficient of each subscale ranged from 0.849 to 0.913. The results are shown in Table 2. All values exceeded the standard of 0.7 (Nunnally and Bernstein, 1994), indicating good reliability of the scale.

**TABLE 2:** Reliability analysis results (*N* = 177).

Variable	Item number	Cronbach’s alpha
GF	4	0.852
SL	3	0.849
TW	3	0.891
GSE	3	0.913
SE	4	0.911
ESD	4	0.893

The design of the scale was based on the mature scale of existing studies, which has good content validity. AMOS 23.0 was used for confirmatory factor analysis, and the goodness of fit index of the six-factor model was good (χ^2/^df = 2.085, CFI = 0.936, GFI = 0.838, IFI = 0.937, TLI = 0.922, and RMSEA = 0.079) (Arbuckle, 2003). The CR values of all variables were greater than 0.7, and the AVE values were greater than 0.5, indicating that the scale had good convergence validity (Fornell and Larcker, 1981). The square root of AVE of each variable was greater than the correlation coefficient between this variable and all other variables, indicating that the scale had good discriminative validity (Fornell and Larcker, 1981).

### Descriptive Statistics and Correlation Analysis

The mean value, standard deviation and correlation coefficients of all variables are shown in Table 3. The overall level of the virtual simulation game course learning experience was higher. The average values of system goal and feedback, selectivity and team cooperation experience exceeded 6 points. The general self-efficacy level was relatively low at only 5.375. The average value was the lowest of all variables, and the standard deviation was the largest of all variables, indicating that students perceived the game as challenging. The perception difference between students was also large. In the course, students’ overall investment was high, and the students also agreed with the effectiveness of the course in improving entrepreneurial skills. As shown in Table 3, all variables were significantly correlated (*P* < 0.001). The largest correlation coefficient with entrepreneurial skill development was students’ experience of “goals and feedback” in the game, followed by students’ general self-efficacy.

**TABLE 3:** Descriptive statistics and correlation coefficients of each variable (*N* = 177).

	GF	SL	TW	GSE	SE	ESD	CR	AVE
GF	0.772						0.855	0.596
SL	0.699***	0.802					0.843	0.643
TW	0.500***	0.469***	0.875				0.906	0.765
GSE	0.603***	0.613***	0.406***	0.892			0.921	0.795
SE	0.589***	0.573***	0.524***	0.717***	0.852		0.913	0.726
ESD	0.764***	0.723***	0.586***	0.748***	0.736***	0.826	0.896	0.683
The average	6.018	6.075	6.465	5.375	5.713	5.970		
The standard deviation	0.731	0.811	0.646	0.979	0.972	0.827		

The diagonal figures are the square root of AVE of each variable, *** means P < 0.001.

The independent sample *T*-test for gender shows that there were no significant differences between boys and girls in the level of learning experience, learning engagement and perceived entrepreneurial skill development.

### Hypothesis Testing

**FIGURE 1:**
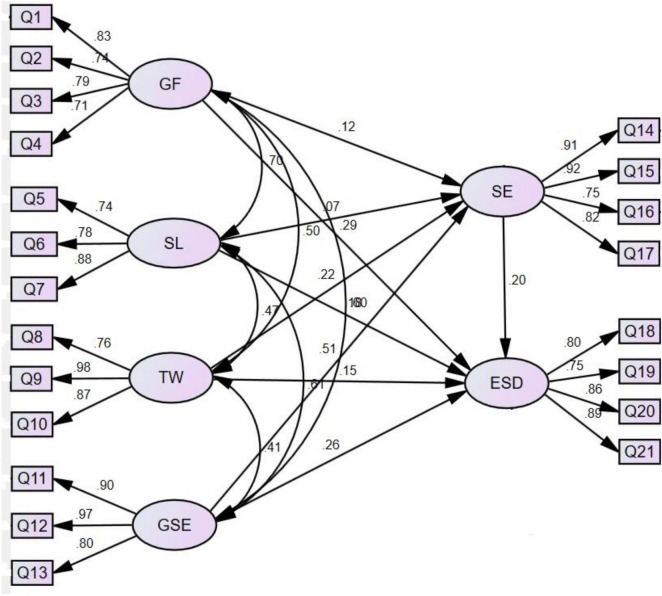
Path analysis results.

AMOS 23.0 was used to test the hypotheses, and the results are shown in Figure 1 and Table 4. The model fitting index was good (χ^2/^df = 2.085, CFI = 0.936, GFI = 0.838, IFI = 0.937, TLI = 0.922, and RMSEA = 0.079) (Arbuckle, 2003). As shown in Table 4, GF had no significant effect on SE (β = 0.118, *P* > 0.05) and SL on SE (β = 0.072, *P* < 0.01), and H2a was not supported. TW had a significant positive effect on SE (β = 0.223, *P* < 0.001), and H2b was verified. GSE had a significant positive effect on SE (β = 0.510, *P* < 0.001), and H2c was verified. GF had a significant positive effect on ESD (β = 0.294, *P* < 0.001) and SL had a significant positive effect on ESD (β = 0.175, *P* < 0.05), and H3a was verified. TW had a significant positive effect on ESD (β = 0.146, *P* < 0.05), H3b was verified. GSE had a significant positive effect on ESD (β = 0.260, *P* < 0.001), and H3c was verified. SE had a significant positive effect on ESD (β = 0.199, *P* < 0.05), and H4 was verified.

**TABLE 4:** Path analysis and hypothesis testing results.

The path	Non-standardized path coefficient	Normalized path coefficient	S.E.	C.R.	Significance level
GF - SE	0.176	0.118	0.145	1.213	0.225
SL - SE	0.090	0.072	0.12	0.749	0.454
TW - SE	0.356	0.223	0.106	3.369	***
The GSE - SE	0.576	0.510	0.094	6.155	***
GF - ESD	0.374	0.294	0.11	3.398	***
The SL to ESD	0.186	0.175	0.087	2.132	*
TW - ESD	0.199	0.146	0.079	2.517	*
GSE to ESD	0.250	0.260	0.076	3.298	***
SE to ESD	0.170	0.199	0.067	2.539	*

*means P < 0.05, ***means P < 0.001.

## Discussion

### Research Conclusion

Based on the 3P learning process theoretical model of Biggs (1993), for the virtual simulation game in entrepreneurship education, college students’ learning experience was taken as the pre learning variable, students’ engagement (SE) was taken as the learning process variable and entrepreneurial skill development (ESD) was taken as the learning outcome variable to explore the action mechanism of entrepreneurial learning experience on college students’ entrepreneurial skill development. The following conclusions were drawn:

(1) In the virtual simulation game course, students’ learning experience includes four dimensions of “goal and Feedback (GF),” “selectivity (SL),” “teamwork (TW),” and “general self-efficacy (GSE)” experience, among which GF and SL refer to the experience of game design itself. The student experience of virtual simulation games is a comprehensive experience, covering at least the game design itself, the team, and self-efficacy.
(2) GF (β = 0.118, *P* > 0.05) and SL (β = 0.072, *P* < 0.01) had no significant effect on SE, TW (β = 0.223, *P* < 0.001) and GSE (β = 0.510, *P* < 0.001) had significant positive effect on SE. An observation can be made that “objectives and feedback” and “selectivity” of game design do not have a direct impact on students’ engagement, but students’ self-efficacy in games is the most significant indicator of students’ engagement, and their experience of team cooperation atmosphere is also a significant indicator of students’ engagement.
(3) GF had significant positive effects on ESD (β = 0.294, *P* < 0.001), SL on ESD (β = 0.175, *P* < 0.05), TW on ESD (β = 0.146, *P* < 0.05), and GSE on ESD (β = 0.260, *P* < 0.001). Thus, “goals and feedback” and “selectiveness” of game design have a direct and significant positive impact on students’ entrepreneurial skill development, while teamwork experience and general self-efficacy not only have a direct and significant positive impact on students’ entrepreneurial skill development, but also indirectly affect the entrepreneurial skill development through students’ engagement degree.
(4) SE has a significant positive effect on ESD (β = 0.199, *P* < 0.05), indicating that the more time, energy and emotion students invest in virtual simulation game courses, the more they perceive their entrepreneurial skills, such as decision-making ability, problem-solving ability, innovation ability and teamwork ability, to be improved.
(5) There is no significant difference between students of different genders in learning experience, learning engagement and perceived improvement of entrepreneurial skills in virtual simulation game courses.

### Theoretical Significance

Firstly, focusing on the student experience in virtual simulation game courses, multiple dimensions were comprehensively considered in the present study, such as game design, team cooperation and student self-efficacy, and a structural equation model was constructed to test their relationship with student engagement and entrepreneurial skill development, which is a significant innovation. Several existing studies have only focused on games themselves (Almeida and Buzády, 2019; Yen and Lin, 2020; Capelo et al., 2021; Zulfiqar et al., 2021), teamwork (Arias-Aranda and Bustinza-Sánchez, 2009; Lohmann et al., 2018; Buil et al., 2020; Thanasi-Boçe, 2020), and students’ personal traits (Eggers et al., 2017; Alstete and Beutell, 2019; Charrouf and Janan, 2019; Hernández-Lara et al., 2019; Buil et al., 2020), lacking a comprehensive study design.

Secondly, based on the theoretical framework of learning process, the influencing factors of college students’ entrepreneurial skills development was investigated in the present study, which is innovative. Existing studies on the effect of virtual simulation game courses on college students’ entrepreneurial learning results have generally been based on the planned behavior theory (Newbery et al., 2016; Zulfiqar et al., 2018, 2021), the technology acceptance model (Zulfiqar et al., 2018, 2021) and Flow theory (Choi and Kim, 2004; Yen and Lin, 2020), which tend to limit the perspective to the game system itself. As such, there is a failure to fully present the complete chain of “input-process-result” and its mechanism of entrepreneurial simulation learning. In the present study, Biggs (1993) 3P learning process theoretical model was applied to the field of entrepreneurship education, the relationship between entrepreneurial learning experience, student engagement and entrepreneurial skill development was explored, and the path mechanism of entrepreneurship education influencing the outcome of entrepreneurial learning was deepened and expanded from the perspective of student learning. Such factors are crucial for advocating student-centered entrepreneurship education (Robinson et al., 2016).

Thirdly, the present study has considerable theoretical significance for clarifying the mechanism through which game design influences learning outcomes. Previous studies on game design has mostly focused on Flow theory (Csikszentmihalyi, 1990), and most of the results support that game design goals, feedback, and selectivity can significantly positively influence students’ flow experience (Choi and Kim, 2004). Notably, there are also unsupported results (Yen and Lin, 2020). However, existing studies have not further explored the direct impact of game design experience on learning results. Empirical research in the present study confirms that although students’ game design experience does not significantly affect their learning engagement, there is a direct and significant positive impact on learning results.

### Practical Significance

In the context of the global COVID-19 pandemic, the present study has considerable practical significance for entrepreneurship education. By introducing online virtual simulation games into entrepreneurship classes, students can learn entrepreneurship through the Internet and develop their entrepreneurial skills. The results of the present study have significant practical implications for teachers in terms of how to better conduct virtual simulation game courses.

First, when introducing virtual simulation games into entrepreneurship courses, teachers should pay attention to whether the design of the game itself meets the teaching objectives, whether the game establishes clear performance evaluation standards, provides rich and clear learning materials, and whether it can provide clear and clear feedback to students after each decision-making operation. Attention should also be paid to whether the game provides different levels of difficulty and different environmental parameters for teachers to adjust settings, so as to provide students with an “optional” competition environment. Such factors can directly affect students’ sense of learning.

Secondly, teachers should pay special attention to the atmosphere of students’ team in the teaching of virtual simulation games. Because students’ experience of the team atmosphere will not only affect students’ engagement, but also directly affect the learning result. Teachers can establish an assessment mechanism and take team participation as an indicator of students’ individual assessment to avoid free-riding. At the same time, for teams with conflicts, teachers need to actively intervene, adjust and encourage the CEO to create a harmonious, democratic and mutual trust team atmosphere.

Thirdly, teachers should pay attention to improving students’ general self-efficacy in the course of virtual simulation games. The results of the present study show that students’ general self-efficacy has the greatest impact on students’ engagement, and is also a significant factor affecting the development of entrepreneurial skills. Teachers need to review relevant professional knowledge before the game, explain the game rules in detail, and test to understand students’ mastery of relevant knowledge and rules. After 1–2 rounds of competition, teachers can also allow students to choose the difficulty level of the competition independently, so as to enhance students’ sense of self-efficacy, and then improve students’ investment and ability, which is consistent with the research conclusions of Alstete and Beutell (2019).

### Research Limitations and Prospects

There were several limitations in the present study. First, the focus was on the three factors of game, team and individual in the course of virtual simulation games, but the factor of teacher may be ignored, which needs to be supplemented in future research. Second, entrepreneurial skill development is used as an evaluation index of learning results in the present study, and future research can be further extended to entrepreneurial intention, entrepreneurial behavior and other indicators. Third, the method of questionnaire survey was adopted to collect cross-sectional data. In the future, qualitative research and experimental research can be used comprehensively to ensure more rigorous research results.

## Data Availability Statement

The raw data supporting the conclusions of this article will be made available by the authors, without undue reservation.

## Author Contributions

QY determined the research theme, research framework, questionnaire design, data analysis method, and was responsible for the finalization of the manuscript. YZ was responsible for literature collation, questionnaire implementation, and draft writing. YL was responsible for the collation and analysis of data. All authors contributed to the article and approved the submitted version.

## Conflict of Interest

The authors declare that the research was conducted in the absence of any commercial or financial relationships that could be construed as a potential conflict of interest.

## Publisher’s Note

All claims expressed in this article are solely those of the authors and do not necessarily represent those of their affiliated organizations, or those of the publisher, the editors and the reviewers. Any product that may be evaluated in this article, or claim that may be made by its manufacturer, is not guaranteed or endorsed by the publisher.
