# Supplementary material for: Study on the Influence Mechanism of Virtual Simulation Game Learning Experience on Student Engagement and Entrepreneurial Skill Development
Source: Front Psychol. 2022 Jan 27;12:772157. doi: 10.3389/fpsyg.2021.772157 (PMC8829425; doi:10.3389/fpsyg.2021.772157)
Supplement: Supplementary file 1 [file Data_Sheet_1.docx]

APPENDIX A. QUESTIONNAIRE ITEMS OF THE STUDY

Dear students,

Thank you for your active participation in this course. At the end of the course, please fill in the following questionnaire according to your own experience. The purpose of this survey is academic research and to better improve teaching, and is not for profit. The questionnaire should be filled in anonymously to respect personal privacy. A score of 1 means strongly disagree, and a score of 7 means strongly agree.

I. Please comment on your learning experience of the course:

| Items | Options |
| --- | --- |
| Q1: The system provided enough information for me to define the goals of this simulation business game | 1 2 3 4 5 6 7 |
| Q2: The system provides the information necessary to achieve the goals of the game | 1 2 3 4 5 6 7 |
| Q3: I know the educational goal of the game | 1 2 3 4 5 6 7 |
| Q4: At the end of each quarter, the system will give feedback to my team's business results | 1 2 3 4 5 6 7 |
| Q5: The game offers different difficulty levels to choose from | 1 2 3 4 5 6 7 |
| Q6: The game offers different macro environment parameters to choose from | 1 2 3 4 5 6 7 |
| Q7: The game gave me the opportunity to make different business strategy choices | 1 2 3 4 5 6 7 |
| Q8: My team works well together | 1 2 3 4 5 6 7 |
| Q9: Our key decisions are made by the whole team | 1 2 3 4 5 6 7 |
| Q10: I have a better understanding of the simulation system through the interaction of team members | 1 2 3 4 5 6 7 |
| Q11: I have the necessary expertise to play the game | 1 2 3 4 5 6 7 |
| Q12: I have the required professional ability to play the game | 1 2 3 4 5 6 7 |
| Q13: I have the computer power required to play the game | 1 2 3 4 5 6 7 |

ⅱ. Please evaluate your investment in the course:

| Items | Options |
| --- | --- |
| Q14: I try to apply what I have learned in entrepreneurship and business management to decision-making | 1 2 3 4 5 6 7 |
| Q15: When I play business simulation games, I am happy | 1 2 3 4 5 6 7 |
| Q16: I think this simulation game is very interesting | 1 2 3 4 5 6 7 |
| Q17: I try to do my best in simulation games | 1 2 3 4 5 6 7 |

ⅲ. Please comment on your entrepreneurial skill development in the course:

| Items | Options |
| --- | --- |
| Q18: The game improved my decision-making ability | 1 2 3 4 5 6 7 |
| Q19: The game has improved my team spirit | 1 2 3 4 5 6 7 |
| Q20: The game improved my ability to apply entrepreneurial knowledge to practice | 1 2 3 4 5 6 7 |
| Q21: The game has improved my ability to innovate in a new environment | 1 2 3 4 5 6 7 |

Q22: Your gender is: □ Male □ female
